# Does Weight-Cycling Influence Illness Beliefs in Obesity? A Gender-Sensitive Approach

**DOI:** 10.1155/2021/8861386

**Published:** 2021-08-21

**Authors:** Svenja Prill, Carmen Henning, Stefanie Schroeder, Sabine Steins-Loeber, Jörg Wolstein

**Affiliations:** ^1^Department of Pathopsychology, University of Bamberg, Bamberg 96047, Germany; ^2^Department of Clinical Psychology and Psychotherapy, University of Bamberg, Bamberg 96047, Germany

## Abstract

Obesity is classified as a chronic disease. Women and men seem to face different obstacles in their attempts to overcome one of the most challenging tasks in the treatment of this disease, namely, weight reduction maintenance. The Common-Sense-Model (CSM) is mainly used to improve the understanding of self-regulation and health behaviour in chronic diseases but has yet to be explored for obesity. This paper applies the CSM to obesity, focussing on the construct of illness representations, which is the basis of health behaviour according to the CSM. A sample of *n* = 356 women and *n* = 77 men with obesity was investigated to assess the extent that illness representations in obesity are shaped by experiences of weight-cycling and the extent that gender influences their quality. Our results show that the representations of timeline and consequences as well as the emotional representation are particularly influenced by weight-cycling, especially in men. On average, women showed more maladaptive illness representations than men. These findings not only contribute to a better applicability of the CSM in obesity, but also emphasize the importance of gender in obesity research and interventions.

## 1. Introduction

Obesity is defined as the excessive accumulation of body fat and is considered a chronic disease by the World Health Organization [WHO] [1]. In Germany, 23% of women and 24% of men are obese [2]. According to the S3-guidelines of the German Obesity Society [3], treatment pursues the two primary goals of weight reduction and its maintenance. Research shows that maintenance can be particularly challenging [4] and that physical and mental health outcomes of obesity vary as a function of gender [5–7]. Still, gender has been insufficiently investigated in obesity research and interventions [7, 8]. Consequently, obesity poses a great challenge for national health systems [9] because of the resulting physical [9, 10] and psychological [11–14] health issues. To counteract the problem, a more individualized treatment approach has been recommended [3]. As a theoretical framework for conceptualising such an approach, Breland, Fox, Horowitz, and Leventhal [15] proposed applying the Common-Sense-Model [CSM] to obesity, a model often used in other chronic diseases to specify mechanisms that influence the course of the disease and treatment outcomes [16]. Notwithstanding its promising utility, the CSM has hardly been explored in obesity. To date, only individual aspects or associated constructs of the CSM are investigated in relation to obesity, but findings have not been classified within the model and no explicit research has been carried out on the applicability of the CSM in relation to obesity [15]. This paper aims to expand knowledge about the CSM for obesity by focussing on the key constructs and the individual beliefs about one's illness and by investigating how people with obesity struggle with maintaining weight, with an emphasis of a gender-sensitive approach.

The CSM is subject to constant development and has recently been reviewed and expanded in an extensive meta-analysis [16]. The model primarily conceptualises processes that underlie an individual's handling of health-threatening stimuli and diseases. Somatic experiences and experiences regarding physical and cognitive functioning, also known as “illness stimuli,” are assumed to be automatically assessed [17, 18]. If a stimulus implies a deviation from a subjectively healthy normal state, it is evaluated as a state of disease, which is then conceptualised as a subjective illness representation [SIR]. An SIR can be divided into an interacting cognitive and emotional representation. The emotional representation refers to the affective state caused by the stimulus, meaning the changed health status. The cognitive representation refers to SIRs in the narrower sense and comprises the following five dimensions: (1) identity, i.e., the label of the illness and symptoms associated with it and causes to which an individual attributes to his or her disease; (2) timeline, i.e., whether the illness is assumed to be acute or chronic and whether its occurrence is perceived as constant or cyclical; (3) social, cognitive, and physical consequences associated with the illness; (4) sense of coherence, i.e., the extent to which individuals can explain the disease to themselves; and (5) subjective sense of control regarding treatment by others as well as personal actions. The dimensions of identity and causes are not the subject of this study and will not be addressed further. Congruent with an SIR, a specific health behaviour ensues, which in turn is evaluated in terms of its effectiveness, meaning the nature of the SIR influences the handling of the illness and therefore the state of health. This outcome in turn can influence the SIR [18]. The entire process is hereby embedded in an individual and social context, which can influence both the development of the SIR and the evaluation process. An SIR is therefore not a static construct but can be modified. However, the authors also emphasized that health behaviours congruent to an SIR quickly turn into automated processes, which can be problematic, especially when trying to change maladaptive behaviours.

So far, the CSM has been applied mainly to chronic diseases such as cardiovascular diseases, diabetes, and cancer [16]. However, Breland and colleagues [15] argued for a clinical application of the CSM, especially for obesity, because it draws attention to automated health-relevant processes. Consequently, the CSM is suitable as a theoretical framework for changing and interrupting automated eating patterns [15]. To be able to apply the CSM in the treatment of obesity, an improved understanding of the individual SIR should be gained. According to the authors, this individualized understanding and a subsequent active consideration of SIRs in treatment can lead to a sustainable change in behaviour, including long-term weight reduction.

In such a model, the individual dietary behaviour can be understood as an illness stimulus in obesity. Individuals with a higher body mass index [BMI] are more likely to try dieting [19]. However, only one-third of all individuals with obesity are able to maintain a weight reduction of 10% for one year, and only 12% report a long-term weight-loss maintenance over five years [20]. To achieve the sustainable behavioural change crucial for long-term weight reduction, a greater consideration of individual factors has been recommended [4, 15].

Weight-cycling often includes repeated dieting attempts and is characterized by a weight regain after such an attempt [3]. Currently, no uniform definition exists that stipulates the cycle frequency or absolute or percentage weight loss and gain that must occur to be classified as weight-cycling [21, 22]. Nevertheless, evidence has shown that particularly people with obesity experience increased weight-cycling, with renewed weight gain often exceeding initial weight loss. Thus, weight-cycling seems to be positively associated with the severity of obesity [23]. Indeed, the number of dieting attempts appears to be a relevant risk factor in the treatment of obesity. In a comprehensive meta-analysis, the number of attempts was identified as the only significant (negative) predictor of successful weight reduction. That is, the greater the number of attempts, the lower the success rate [24].

Given the prevalence of weight-cycling in people with obesity [19], it needs to be considered as an illness stimulus in the CSM of obesity. Weight-cycling is a direct somatic experience because a change in weight also implies a direct somatic change of body weight. In addition, physical limitations resulting from excessive weight, for example, in movement or breathing, can be reduced by weight reduction. Furthermore, if weight reduction fails, obesity consequently will persist along with the associated physical health risks [9, 10]. However, the extent that weight-cycling in individuals with obesity is a direct risk factor is still unclear [22, 25]. The repeated failure to lose weight represents a great psychological burden for those affected. Research has shown that the quality of life improves with weight reduction, just as it worsens with weight regain [26]. In addition, the expectations associated with weight reduction repeatedly result in disappointment. A higher vulnerability towards experiences of failure could manifest in, for example, feelings of helplessness and increased negative attributions or a negative self-image [24]. Frustration as a result of failure and the simultaneous reduction of efforts required to maintain dietary and physical activity could lead to less success in reducing weight [27], which closes the circle.

Leventhal [28] criticized the operationalisation of the CSM, stating that among other aspects the model neglects the individual context, which can have a large impact on the parameters of the CSM. The present study addresses this point of criticism by considering gender as a relevant factor. Evidence shows that consequences of obesity can depend on the gender of the individual. For example, being a female can be seen as a general risk factor concerning the development of mental illness [29]. Additionally, quality of life is more restricted in women with obesity, especially with regard to mental functioning, although the processes leading to an impaired quality of life seem to differ in women and men with obesity [5]. Despite numerous studies reporting gender differences in obesity, these findings have drawn little attention [7], and overall, research on gender differences in the relationship of obesity and mental health has been neglected for too long [29].

Different SIRs should be considered as one possible explanation because the experiences shaping SIRs clearly differ in women and men with obesity, as well as differences in dietary behaviour. Overall, women with obesity make more frequent attempts to lose weight [19, 30]. This higher frequency may be due to different weight-related attitudes [30]. For example, men who are overweight or obese are more satisfied with their weight, but their weight-perception is less accurate compared to women in the same condition. Nevertheless, women seem to be less successful in losing weight, although they try harder [30]. One possible explanation is a lower confidence in their ability to lose weight [31]. Despite these findings, a closer look at the success rates in weight loss and its maintenance reveals a very heterogeneous picture [32]. Robertson and colleagues [8] argued that men are underrepresented in studies on weight loss and maintenance, which may explain the heterogeneity. Crane et al. [33] observed in a randomized controlled trial that women and men differ mainly in their motivation to lose weight. In this respect, the findings of Crane and colleagues [33] imply that, for successful and long-term weight reduction, greater consideration of individual gender-specific beliefs and attitudes is central, supporting the plea for gender-specific designs of weight-loss programmes [8]. In conclusion, women and men with obesity not only have different experiences concerning their illness, but also seem to react to these experiences differently. These differences also apply to dietary behaviour.

## 2. Method

### 2.1. Research Questions and Hypotheses

In a CSM for obesity, weight-cycling could be understood as an illness stimulus that influences the development of a SIR, as stated above. Assuming an effect of weight-cycling on the SIR and a higher frequency in individuals with obesity [19], the effect is not present in all cases. This study will therefore examine the extent to which SIRs differ in individuals with obesity, depending on the presence of weight-cycling in their dietary history.

 
*Research Question 1:* Does the SIR in obesity differ between individuals with and without weight-cycling?

Obesity is already defined as a chronic illness [1]. If the weight remains the same after repeated, unsuccessful dieting attempts, a chronic rather than an acute timeline is implied. Moreover, the illness returns repeatedly with weight-cycling such that a cyclical timeline becomes evident. Obesity is further associated with serious physical [9, 10] and psychological consequences [11–13], and weight-cycling results in an improvement or deterioration in quality of life [26]. Consequently, if dieting attempts are unsuccessful, not only does health risks remain, but a quality of life deterioration is directly perceptible. Individuals with obesity and weight-cycling should therefore expect more serious consequences of their illness. Repeated negative experiences with weight reduction also lead to increased negative affect in relation to the illness such that the emotional representation is more pronounced.

 
*Hypothesis 1a:* Individuals with weight-cycling experience a more chronic and cyclical timeline, resulting in more serious consequences, including a more pronounced emotional representation than individuals without weight-cycling.

Weight-cycling also implies a reduced subjective sense of control, characterized by insufficient efforts, ineffective strategies, and unsuccessful dieting. The frustration that comes with such failures has further implications. Consequently, the illness can become less explainable because the treatment considered to be effective has no desired effect.

 
*Hypothesis 1b*: Individuals with weight-cycling feel less personal control and less treatment control and have a lower sense of coherence than individuals without weight-cycling.

The given hypotheses may not apply equally to women and men with obesity. Firstly, they differ in the examined predictor of the SIR because women tend to diet more often than men [19, 30]. Secondly, determinants of the SIR may differ between women and men, for example, in the comorbidities associated with obesity [5, 6, 29] or weight-related attitudes [30]. Thus, this study also raises the question of whether women and men with obesity differ in their SIR.

 
*Research Question 2*: Do women and men with obesity differ in their SIR?
 
*Hypothesis 2*: Women and men with obesity differ in their SIR.

### 2.2. Data Collection

The data collection took place within the I-GENDO study (the I-GENDO study takes place in cooperation with the University of Bamberg and the LWL University Hospital of the Ruhr University Bochum). The aim of the study is to develop a gender-sensitive app that takes individual psychological factors into account to improve the effectiveness of weight reduction measures in obesity. For further information, please contact the study management in Bamberg (sabine.steins-loeber@uni-bamberg.de; joerg.wolstein@uni-bamberg.de) or Bochum (stephan.herpertz@rub.de). The study was done via online assessment from April 2017 until March 2018. The participants had the chance to win one of four €50 shopping coupons. The link to the online survey was publicly available and was disseminated via e-mails to self-awareness groups, nutrition experts, and clinical institutions and advertised on social networks. This study is a secondary analysis of the cross-sectional data collected in the first step of the I-GENDO study. The total sample comprised 962 participants of which 524 were excluded. Exclusion criteria for the present study were missing values on one or more of the following variables: gender, height, weight, SIR, and frequency of dietary attempts. In addition, the participant needed to have a BMI of at least 30 kg/m^2^ as well as a clear consent to participate in the study.

### 2.3. Measures

*Sociodemographic Data.* The participants were asked to indicate their gender (*male or female*) and age. Furthermore, in a single-choice format, the highest level of educational and vocational training certificate was requested as well as their type of employment. For each of the three categories, individuals were classified into two groups: having at least a lower secondary education (or not); having a vocational training certificate (or not); being employed at least marginally (or not).

*Weight and Height.* The current body weight and height were requested via self-report. On the basis of these criteria, the BMI could be calculated to classify each individual according to the WHO [1] as obesity level I (BMI: 30.00–34.99 kg/m^2^), obesity level II (BMI: 34.99–39.99 kg/m^2^), and obesity level III (BMI ≥ 40.00 kg/m^2^).

*Weight-Cycling.* The participants were asked to indicate the number of previous dieting attempts (*never; 1 to 5 times; 6 to 9 times; 10 times or more*). All individuals in the category “10 times or more” were considered weight-cyclers; all individuals with fewer than 10 attempts were considered to be non-weight-cyclers.

*Subjective Illness Representation.* The SIR was measured using the German version of the Illness Perception Questionnaire-Revised [IPQ-R] [34, 35]. With an internal consistency of *α* = 0.70 to *α* = 0.87, the IPQ-R proves to be a reliable measurement [36]. In the current study, the subscales “acute-chronic timeline,” “cyclical timeline,” “consequences,” “personal control,” “treatment control,” “sense of coherence,” and “emotional representation” were evaluated. They comprise a total of 32 items, which are rated on a 5-point Likert scale from 1 (“*disagree very strongly*”) to 5 (“*agree very strongly*”). In the present sample, internal consistency was from *α* = 0.65 to *α* = 0.89 in the overall sample, from *α* = 0.66 to *α* = 0.88 in the female subsample, and from *α* = 0.60 to *α* = 0.92 in the male subsample. An overview of all values is given in Table S1 of the Supplementary Material. For each subscale, a total value can be calculated, ranging from 5 to 25 points on the scales “acute-chronic timeline,” “consequences,” “sense of coherence,” and “emotional representation” and from 5 to 20 points on the scales “cyclical timeline,” “personal control,” and “treatment control.” Higher values imply a stronger expression of the assumption on which the corresponding representation is based.

### 2.4. Statistical Analyses

SPSS v. 26.0 was used for all analyses. All hypotheses were examined in the total sample and separately for the subsamples of women and men. All hypothesis tests were conducted with an alpha level of *α* = 0.05. To characterize the sample, an additional *χ*^2^ test was used to calculate the extent to which women and men differ in obesity level and weight-cycling. Cramer's *V* was given as the effect size for women and the *φ*-coefficient for men. For all other hypotheses, Hedges *g* was calculated as the effect size because this measure has proven to be particularly useful for different sample sizes [37]. The effect-size-calculator by Hemmerich [38] was used for calculations.

To test hypotheses 1a and 1b, a one-sided *t*-test for independent samples was computed. The part of the male subsample that was considered weight-cyclers fell below the required sample size of *n* ≥ 30 [39]. According to Hayes [40], however, the *t*-test is very robust against violations of the normal distribution assumption, which is why a separate calculation of the group differences using a nonparametric procedure was omitted. Participants classified as non-weight-cyclers were assigned the value 0, and weight-cyclers were assigned the value 1. Since hypotheses 1a and 1b were directional hypotheses, the one-sided *p* value was calculated according to equation 1 in the case of hypothesis-conforming mean differences and according to equation 2 in the case of contradicting mean differences. According to the coding of the groups, a significant *t* value meant that the respective dimension of the SIR was deemed statistically significant to a greater or lesser extent among weight-cyclers compared to non-weight-cyclers. To examine possible gender differences in terms of the SIR (hypothesis 2), a two-sided *t*-test for independent samples was conducted. The normal distribution was not checked because both subsamples had a sample size of *n* > 30 [39].

## 3. Results

The final sample comprised *N* = 433 individuals (*n* = 356 women, *n* = 77 men). The overall sample was on average *M* = 42.50 (SD = 10.80) years old, whereby the average age of women was *M* = 41.08 (SD = 10.38) and the age of men was *M* *=* 49.06 (SD *=* 10.32). All men and almost all women (98%) had at least a lower secondary education (missing values on the variable “school leaving certificate” were found in 8 cases (7 women, 1 man)), and 89% of the women as well as 90% of the men had a vocational training certificate (missing values on the variable “vocational training certificate” were found in 8 cases (7 women, 1 man)). Furthermore, 69% of the women and 80% of the men were at least marginally employed (missing values on the variable “employment” were found in 3 cases (2 women, 1 man)), 26% of the women and 10% of the men were not working (anymore), and 5% of the women as well as 9% of the men were unemployed. We found a significant gender difference in the distribution of the obesity level (*V* = 0.30) and in weight-cycling (*φ* = −0.26) (see Table 1). A detailed overview of the values on the IPQ-R is given in Table 2.

### 3.1. Subjective Illness Representations and Weight-Cycling

**Figure 1:**
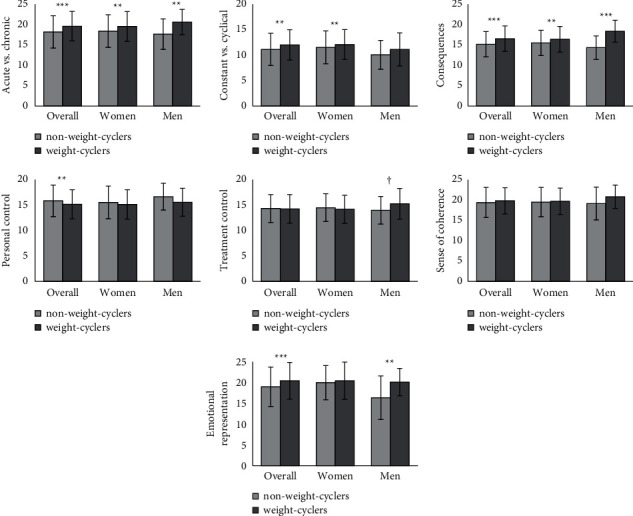
Expression of the SIR in weight- and non-weight-cyclers in the overall sample as well as female and male subsamples (for numerical values see Table 3). The error bars represent the standard deviation of measurements of the SIR for weight- and non-weight-cyclers. *Note.p* value (one-sided): ^†^marginally significant, ^*∗*^*p* < 0.05, ^*∗∗*^*p* < 0.01, ^*∗∗∗*^*p* < 0.001; weight-cyclers: 10 or more diet attempts (overall *N* *=* 212, women *n* *=* 196, men *n* *=* 16), non-weight-cyclers: fewer than 10 or no diet attempts (overall *N* *=* 221, women *n* *=* 160, men *n* = 61).

An overview of all results is given in Figure 1. For detailed numerical values, see Table 3.

In the overall sample, significant differences between weight-cyclers and non-weight-cyclers were found with small effect sizes. Compared to non-weight-cyclers, weight-cyclers assumed a more chronic as well as cyclical timeline, presumed more serious consequences, felt less personal control over their illness, and showed a more pronounced emotional representation. No significant differences were found in treatment control and sense of coherence. Hypothesis 1a could thus be accepted in full and hypothesis 1b in part.

Female weight-cyclers and non-weight-cyclers also differed with small effect sizes. Female weight-cyclers assumed a more chronic as well as cyclical timeline and presumed more serious consequences. No significant differences were found in personal and treatment control, sense of coherence, and emotional representation. Hypothesis 1a could thus be accepted in part for the female subsample, whereas hypothesis 1b was rejected.

In the male subsample, significant differences were found with large effect sizes. Male weight-cyclers assumed a more chronic timeline, presumed more serious consequences, and showed a more pronounced emotional representation. No significant differences were found regarding the assumption of a constant versus a cyclical timeline, personal control, and sense of coherence. A marginally significant difference was found between male weight-cyclers who felt more in control over their treatment compared to non-weight-cyclers. However, this finding was contrary to the hypothesis. Thus, hypothesis 1a was partially accepted for the male subsample and hypothesis 1b was rejected.

### 3.2. Gender Differences in Subjective Illness Representations

Significant differences in the SIR of women and men were found with small to medium effect sizes. For detailed numerical values, see Table 2. Compared to men, women assumed a more cyclical timeline, presumed more serious consequences, felt less personal control, and showed a more pronounced emotional representation. No gender differences were found in the assumption of an acute versus a chronic timeline, treatment control, and sense of coherence. Hypothesis 2 could thus be accepted in part.

## 4. Discussion

This paper aimed to increase the knowledge about a CSM in obesity, focussing on the emergence of the SIR, an area that has not yet been studied for obesity. The illness stimulus underlying the SIR [17, 18] was operationalised as weight-cycling. Additionally, possible gender differences in the SIR were assessed and thus Leventhal' [28] call for greater consideration of individual factors in the CSM and the call for more gender-sensitive research on obesity [7] were addressed. In summary, weight-cycling impacts the perceived severity of consequences for women and men with obesity. In addition, weight-cycling is especially relevant for men in terms of emotional representation. Further gender differences are evident in the overall representation of timeline, consequences, and personal control as well as the emotional representation.

In line with the hypothesis 1a, individuals with obesity and weight-cycling are more likely to perceive the timeline of obesity as chronic and cyclical. The results suggest that weight-cycling indicates a persistent weight and thus obesity is assumed to be persistent. Individuals with weight-cycling seem to consider obesity as an illness, and the timeline and symptoms of the illness are perceived to be subject to fluctuations. However, this assumption only applies to women with obesity, not men. These gender differences may be a result of gender-specific dietary behaviour, given that women with obesity diet more often [19, 29], as confirmed in this study, which increases the likelihood of weight-cycling [22]. In light of this finding, weight-cycling may be a more salient stimulus of the timeline presentation in women than in men. In addition, the women in this sample showed a more pronounced overall representation of a cyclical timeline. Again, this may be traced back to a gender-specific dietary history.

The results also confirm that individuals with obesity and weight-cycling assume more serious consequences, regardless of gender. This is in line with the assumption of Breland and colleagues [15] that dietary behaviour is associated with a more pronounced representation of consequences, although in this study dietary behaviour is assumed to be an illness stimulus, not a health behaviour. Also, women with obesity, in general, assume more serious consequences. Being female can be seen as risk factor for obesity-related consequences such as weight-based discrimination or self-stigmatisation [29]. As a result, women in this sample may not only have perceived consequences to be more serious but may have even experienced more negative consequences.

A more complex picture emerged from the analysis of gender and weight-cycling in affective states and its effects on emotional representations. Only male weight-cyclers showed more negative affect towards obesity, consistent with the hypothesis that repeated experiences of failure can lead to an increase in negative emotions. Men with obesity and weight-cycling thus seem to be more emotionally burdened than men without the illness, and the weight-cycling seems to play a more important role in the affective states associated with obesity in men than in women. One possible explanation could be gender differences in body perception. Men tend to underestimate their body weight, resulting in a less accurate body-related self-perception [41]. According to the author, health behaviour, such as dieting, is only implemented when weight is perceived as a health risk. The more pronounced emotional representation in male weight-cyclers may thus be a result of a greater awareness of body weight and associated risks. This awareness may lead to more negative affect towards one's weight, which had previously been shielded by the protective function of a biased perception. In contrast, women with obesity generally show a more pronounced emotional representation than men. Studies have reported that women are more often disappointed in obesity treatment, which may be reflected in the emotional representation. For example, women with obesity have higher weight-loss expectations and aim for greater weight reduction than men, even after completing a weight-loss program [42]. They also show a greater discrepancy between their current and desired weight [43] and are at higher risk of experiencing negative health outcomes such as depression or anxiety [29] and greater body-related shame and guilt [44], which cumulatively may lead to more negative affect.

Whilst hypothesis-conforming effects can be found for the representation of timeline and consequences as well as for the emotional representation, results are less clear for the sense of control and coherence. Weight-cycling is indeed associated with less personal control. However, this association only applies to the total sample. No effects were found in the subsamples of women and men, possibly because of the decrease in sample sizes. Nonetheless, a direct comparison of women and men shows that women with obesity feel less personal control over their illness than men. This difference corresponds to the analogous structure of the SIR [16] given that the representation of consequences and timeline and the emotional representation are more pronounced in the female subsample. If the sense of control is understood as a form of self-efficacy, our result is also consistent with the findings of Bonsaksen et al. [45] who reported that higher scores in the stated representations correlate negatively with self-efficacy in obesity. However, the underlying mechanisms are yet to be determined.

The similarity of treatment control between weight-cyclers and non-weight-cyclers is surprising, given that dieting seems to be less successful for weight-cyclers. However, the question of whether or not the treatment is successful may be attributed more to other causes. Rather than calling the method into question, an explanation based on personal circumstances may be sought. For example, weight gain after dieting is often attributed to emotional causes such as stress, frustration, or emotional exhaustion [46]. Furthermore, repeated weight-loss attempts could imply a certain degree of treatment control because such behaviour suggests a belief that obesity is a modifiable condition. This explanation is congruent with the assumption of Breland and colleagues [15] who assumed that a feeling of control is associated with the onset of weight-loss measures. The explanation also agrees with the tendency of male weight-cyclers to feel more treatment control, contrary to the hypothesis, highlighting the assumption that weight-cycling plays a different role for men than for women, as well as for the emotional representation. In addition, attribution can be a decisive factor because dieting can be seen as a method that requires a high degree of personal control and effort. Effort, in turn, is an internally anchored and causal variable for success or failure in a task [47]. As with effort, the controllability of this cause leads to an internal attribution of failure. Dieting thus appears to be an effective method, at least in the short term, whereas weight gain can be attributed to internal causes such as lack of effort or emotional states [46]. The question of whether gender differences can be explained by different attributional patterns requires further clarification. Finally, the sense of coherence may also depend on other parameters such as weight-cycling. At the core of the related scale is the question of whether or not an individual can explain their illness to themselves. For a deeper understanding of the determinants of the sense of coherence, investigating the causes of disease assumed by those affected would provide useful information.

## 5. Limitations

This study has some limitations that need to be addressed. Firstly, the CSM proposes a causal relationship between an illness stimulus and the SIR. However, the analysed data are cross-sectional rather than longitudinal. Therefore, the extent that weight-cycling determines the expression of the SIR cannot be determined. Secondly, the internal consistency of the subscales of constant versus cyclical timelines and treatment control are problematic [36], as confirmed in this study (see Table S1 in Supplementary Material). The former generally appears to have an ambiguous factor structure, and the latter is not sufficiently distinguishable from the construct of personal control. Therefore, the extent that the measurement of these two subscales is insufficiently accurate cannot be ruled out. Accordingly, the results must be interpreted with caution, especially because the IPQ-R has not yet been generally validated for a sample of individuals with obesity or for individuals with extreme obesity. However, as stated by Breland and colleagues [15], the use of validated instruments to record the SIR is the first step towards using the CSM in a clinical context related to obesity. Further research is therefore necessary. The internal consistency of the present work can already be seen as an important first step in this process, indicating that the IPQ-R is applicable to obesity. Thirdly, the operationalisation of weight-cycling and the classification of participants as weight-cyclers or non-weight-cyclers were only indirectly deduced from the number of self-reported diets carried out. However, the reader should note that no uniform definition of weight-cycling exists and no consensus on the number of weight fluctuations required to qualify as weight-cycling has been reported in the literature [21, 22].

Apart from these methodological considerations, the comparison between women and men on the expression of the SIR also presents limitations because the possibility cannot be entirely ruled out that gender differences are partly due to the BMI. The BMI of the female subsample is significantly higher and lies within the range of a level III obesity, which differs substantially from lower levels of obesity [14, 48, 49] and could thus have impacted the results. However, the subsamples of women and men are quite comparable, which is relevant because variables such as educational background are also associated with obesity [9]. Another possible limitation was the assessment of obesity severity, which was based on self-reported measures of weight and height. However, self-reported height is systematically overestimated by both women and men, whereas weight is systematically underestimated, leading to an underestimation of the BMI calculated from self-reported data compared to direct measures [50]. The conclusions drawn concerning the SIR therefore tend to be valid for individuals with higher levels of obesity, as the calculated BMI suggests. This conclusion is important because individuals with extreme obesity represent a specific subgroup [51]. Finally, many individuals notably were excluded from the study because the participant's weight, height, or number of diets undertaken were not reported.

## 6. Conclusion

This study confirms that weight-cycling affects the SIR in obesity with different implications for women and men. Further gender differences are evident in the overall SIR. As a result, an improved understanding of the individual SIR is recommended [15], and gender must be considered to achieve a clinical application of the CSM in obesity.

In light of the meta-analysis of Hagger and colleagues [16], weight-loss programs applying a CSM for obesity should focus on both directly modifying the SIR and supporting adaptive coping strategies with maladaptive SIRs to promote constructive health behaviour and possible weight-loss maintenance. Targeting the SIR seems promising for the subjective sense of control, given that this SIR is linked directly and indirectly to health behaviour [16]. One approach could be the reinforcement of self-efficacy because this construct correlates not only positively with weight-loss maintenance [4], but also negatively with depressive symptoms and anxiety [36, 52]. Given the fact that women with obesity feel substantially less control over their illness, they should be considered as a risk group. This assertion also applies to interventions for the representation of consequences and the emotional representation because, according to the findings in this study, women with obesity seem to be at higher risk of having more maladaptive SIRs in general. However, working on the perceived severity of consequences and the negative affective states requires a more individual approach. The consequences of high stress-levels (e.g., due to a pronounced representation of consequences or negative affect) on health behaviour depend on whether the individual gets motivated or perceives the arousal as a threat and thus chooses adaptive, problem-focused, or maladaptive coping strategies [16]. A commonly used and promising technique in interventions using the CSM could be reattribution [53]. In using this technique, both female and male weight-cyclers should be considered a specific risk group in terms of consequences. However, practitioners should especially attend to male weight-cyclers because of their tendency to experience negative affect. Finally, when identifying weight-cyclers as a potential risk group, practitioners should also keep in mind that this very risk may be a resource as well. After all, repeated attempts to lose weight also imply motivated behaviour, which should be acknowledged. Channelling this motivation into weight-loss maintenance might augment the likelihood of success.

A conception of possible interventions and further investigation into underlying mechanisms are still pending. However, in general this study supports the necessity of an individual understanding of SIRs in obesity [15] and the need to adopt a gender-sensitive approach [8].

## Figures and Tables

**Table 1:** Characteristics of the total sample as well as female and male subsamples with gender differences in level of obesity and weight-cycling.

	Total sample (*N* = 433)		Women (*n* = 356)		Men (*n* = 77)		*χ* ^2^	d*f*
	M	SD	M	SD	M	SD		
Age	42.50	10.80	41.08	10.38	49.06	10.32		
BMI in kg/m^2^	41.75	9.03	42.49	8.61	38.35	10.15		
Obesity level (%)							37.76^∗∗∗^	2
Level I	26		20		53			
Level II	22		23		18			
Level III	52		57		29			
Weight-cyclers (%)							29.77^∗∗∗^	2
Yes	49		55		21			
No	51		45		79			

*Note.p* value (two-sided), ^*∗∗∗*^*p* < 0.001; weight-cyclers: 10 or more diet attempts, non-weight-cyclers: fewer than 10 or no diet attempts.

**Table 2:** Total values of the IPQ-R and gender differences in subjective illness representations.

	Overall (*N* = 433)		Women (*n* = 356)		Men (*n* = 77)		*t*	d*f*	*p*	*g* [CI]
	M	SD	M	SD	M	SD				
IPQ-R										
Acute vs. chronic	18.91	3.86	19.5	3.86	18.29	3.81	1.58	431	0.12	0.20 [−0.05; 0.45]
Constant vs. cyclical	11.58	3.12	11.85	3.08	10.30	2.93	4.04	431	0.001	0.51 [0.26; 0.76]
Consequences	15.86	3.20	16.01	3.12	15.18	3.30	2.06	431	0.04	0.26 [0.01; 0.51]
Personal control	15.51	3.00	15.31	3.03	16.43	2.68	−2.99	431	<0.001	−0.38 [−0.62; −0.13]
Treatment control	14.29	2.75	14.30	2.18	14.23	2.80	0.19	431	0.85	0.02 [−0.22; 0.27]
Sense of coherence	19.61	3.48	19.63	3.40	19.51	3.86	0.27	431	0.79	0.03 [−0.21; 0.28]
Emotional representation^a^	19.70	4.63	20.25	4.33	17.14	5.12	4.95	101	0.001	0.62 [0.37; 0.87]

*Note.p* value (two-sided); IPQ-R: Illness Perception Questionnaire-Revised, range of the subscales “acute vs. chronic,” “consequences,” “sense of coherence,” and “emotional representation” from 0 to 25, range of the subscales “constant vs. cyclical,” “personal control,” and “treatment control” from 0 to 20; ^a^Welch correction.

**Table 3:** Differences in subjective illness representations depending on weight-cycling.

		Weight-cyclers		Non-weight-cyclers		*t*	d*f*	*p*	*g* [CI]
		M	SD	M	SD				
IPQ-R									
Acute vs. chronic	Overall^a^	19.63	3.61	18.22	3.96	3.86	431	0.001	0.37 [0.18; 0.56]
	Women^a^	19.55	3.65	18.43	4.02	2.75	354	0.01	0.29 [0.08; 0.50]
	Men	20.63	3.12	17.67	3.76	2.89	75	0.01	0.80 [0.23; 1.37]
Constant vs. cyclical	Overall	12.03	2.98	11.14	3.18	3.00	431	<0.001	0.29 [0.10; 0.48]
	Women	12.10	2.95	11.54	3.22	1.71	354	0.09	0.18 [−0.03; 0.39]
	Men	11.13	3.24	10.08	2.84	1.27	75	0.21	0.35 [−0.20; 0.91]
Consequences	Overall	16.56	3.15	15.19	3.11	4.56	431	0.001	0.44 [0.25; 0.63]
	Women	16.41	3.15	15.51	3.12	2.69	354	0.01	0.29 [0.08; 0.50]
	Men	18.38	2.70	14.34	2.91	4.99	75	0.001	1.39 [0.79; 1.99]
Personal control	Overall	15.16	2.87	15.84	3.09	−2.36	431	0.02	−0.23 [−0.42; −0.04]
	Women	15.13	2.88	15.53	3.20	−1.24	354	0.22	−0.13 [−0.34; 0.08]
	Men	15.56	2.76	16.66	2.64	−1.46	75	0.15	−0.41 [−0.96; 0.15]
Treatment control	Overall	14.25	2.79	14.33	2.73	−0.32	431	0.75	−0.03 [−0.22; 1.16]
	Women	14.16	2.76	14.47	2.73	−1.04	354	0.30	−0.11 [−0.32; 0.10]
	Men	15.25	3.00	13.97	2.70	1.65	75	0.10	0.46 [−0.10; 1.02]
Sense of coherence	Overall^a^	19.81	3.21	19.41	3.72	1.18	426	0.24	0.11 [−0.08; 0.30]
	Women	19.72	3.23	19.51	3.61	0.60	354	0.55	0.06 [−0.15; 0.27]
	Men	20.81	2.86	19.16	4.03	1.54	75	0.13	0.43 [−0.13; 0.99]
Emotional representation	Overall	20.44	4.41	18.99	4.73	3.30	431	<0.001	0.32 [0.13; 0.51]
	Women	20.46	4.49	18.99	4.11	1.03	354	0.30	0.11 [−0.10; 0.32]
	Men^a^	20.13	3.28	16.36	5.25	3.55	38	<0.001	0.99 [0.41; 1.56]

*Note.p* value (two-sided), interpretation according to hypotheses (one-sided); weight-cycling: number of previous dieting attempts, weight-cyclers: 10 times or more (overall *N* = 212, women *N* = 196, men *N* = 16), non-weight-cyclers: less than 10 times or none (overall *N* = 221, women *N* = 160, men *N* = 61); IPQ-R: Illness Perception Questionnaire-Revised, range of the subscales “acute vs. chronic,” “consequences,” “sense of coherence,” and “emotional representation” from 0 to 25, range of the subscales “constant vs. cyclical,” “personal control,” and “treatment control” from 0 to 20; ^a^Welch correction.

## Data Availability

The dataset supporting the findings of this paper belongs to the I-GENDO study. Currently, the data are not publicly available but can be obtained upon reasonable request and with permission from the corresponding author, Jörg Wolstein (joerg.wolstein@uni-bamberg.de).

## Supplementary Materials

Supplementary Materials Table S1: detailed overview of the internal consistency of the IPQ-R.
