# Supplementary material for: Does Weight-Cycling Influence Illness Beliefs in Obesity? A Gender-Sensitive Approach
Source: J Obes. 2021 Aug 21;2021:8861386. doi: 10.1155/2021/8861386 (PMC8405317; doi:10.1155/2021/8861386)
Supplement: Supplementary Materials — Table S1: detailed overview of the internal consistency of the IPQ-R. [file 8861386.f1.docx]

**Supplemental Material**

Table S1. Cronbach's α for the Illness Perception Questionnaire - Revised

|  | Items | Total sample | Women | Men |
| --- | --- | --- | --- | --- |
|  |  | (*N* = 433) | (*n* = 356) | (*n* = 77) |
| IPQ-R |  |  |  |  |
| Acute vs. chronic | 5 | .82 | .82 | .80 |
| Constant vs. cyclical | 4 | .66 | .67 | .60 |
| Consequences | 5 | .80 | .77 | .81 |
| Personal control | 4 | .78 | .77 | .78 |
| Treatment control | 4 | .65 | .66 | .61 |
| Sense of coherence | 5 | .73 | .71 | .82 |
| Emotional representation | 5 | .89 | .88 | .92 |
